# The interdependent transport of yeast vacuole Ca^2+^ and H^+^ and the role of phosphatidylinositol 3,5-bisphosphate

**DOI:** 10.1016/j.jbc.2022.102672

**Published:** 2022-11-02

**Authors:** Chi Zhang, Yilin Feng, Adam Balutowski, Gregory E. Miner, David A. Rivera-Kohr, Michael R. Hrabak, Katherine D. Sullivan, Annie Guo, Jorge D. Calderin, Rutilio A. Fratti

**Affiliations:** 1 Department of Biochemistry, University of Illinois Urbana-Champaign, Urbana, Illinois, USA; 2 Center for Biophysics & Quantitative Biology, University of Illinois Urbana-Champaign, Urbana, Illinois, USA

**Keywords:** calcium ATPase, calcium transport, Fab1, lysosome, organellar pH homeostasis, phosphoinositide, PIKfyve, Pmc1, Vcx1, V-ATPase, Vph1, Stv1, AO, acridine orange, CaM, calmodulin, CQ, chloroquine, DMSO, dimethyl sulfoxide, FCCP, carbonyl cyanide-4-(trifluoromethoxy) phenylhydrazone, TRP, transient receptor potential, YPD, yeast extract–peptone–dextrose

## Abstract

Yeast vacuoles are acidified by the v-type H^+^-ATPase (V-ATPase) that is comprised of the membrane embedded V_O_ complex and the soluble cytoplasmic V_1_ complex. The assembly of the V_1_-V_O_ holoenzyme on the vacuole is stabilized in part through interactions between the V_O_ a-subunit ortholog Vph1 and the lipid phosphatidylinositol 3,5-bisphosphate (PI(3,5)P_2_). PI(3,5)P_2_ also affects vacuolar Ca^2+^ release through the channel Yvc1 and uptake through the Ca^2+^ pump Pmc1. Here, we asked if H^+^ and Ca^2+^ transport activities were connected through PI(3,5)P_2_. We found that overproduction of PI(3,5)P_2_ by the hyperactive *fab1*^*T2250A*^ mutant augmented vacuole acidification, whereas the kinase-inactive *fab1*^*EEE*^ mutant attenuated the formation of a H^+^ gradient. Separately, we tested the effects of excess Ca^2+^ on vacuole acidification. Adding micromolar Ca^2+^ blocked vacuole acidification, whereas chelating Ca^2+^ accelerated acidification. The effect of adding Ca^2+^ on acidification was eliminated when the Ca^2+^/H^+^ antiporter Vcx1 was absent, indicating that the vacuolar H^+^ gradient can collapse during Ca^2+^ stress through Vcx1 activity. This, however, was independent of PI(3,5)P_2_, suggesting that PI(3,5)P_2_ plays a role in submicromolar Ca^2+^ flux but not under Ca^2+^ shock. To see if the link between Ca^2+^ and H^+^ transport was bidirectional, we examined Ca^2+^ transport when vacuole acidification was inhibited. We found that Ca^2+^ transport was inhibited by halting V-ATPase activity with Bafilomycin or neutralizing vacuolar pH with chloroquine. Together, these data show that Ca^2+^ transport and V-ATPase efficacy are connected but not necessarily through PI(3,5)P_2_.

The homeostasis of eukaryotic cells requires the active transport of elements across membranes against concentration gradients. In neurons, action potentials at the presynaptic cleft trigger the influx of Ca^2+^, which in turn facilitates the fusion of synaptic vesicles with the plasma membrane to release neurotransmitters (1). Other cellular gradients include the accumulation of H^+^ in the lysosome to acidify the organelle and promote the activity of luminal hydrolases, and the storage of Ca^2+^ in the endoplasmic reticulum to regulate Ca^2+^-dependent signaling (2, 3).

In *Saccharomyces cerevisiae*, H^+^ and Ca^2+^ ions are oppositely transported across the plasma membrane by the P2 type H^+^-ATPase Pma1 and voltage-gated Ca^2+^ channel Cch1/Mid1, respectively (4, 5). The yeast vacuole differs from the plasma membrane in that the organelle uses ATP hydrolysis to pump both H^+^ and Ca^2+^ ions into the vacuole lumen. The V-ATPase pumps H^+^ into the vacuole, while Ca^2+^ is transported by the Ca^2+^-ATPase Pmc1. Ca^2+^ is also taken into the vacuole lumen through the Ca^2+^/H^+^ (K^+^/H^+^) antiporter Vcx1 (6, 7, 8). Vacuoles can rapidly take up Ca^2+^ after high cellular uptake under stress conditions and can store Ca^2+^ at micromolar concentrations, most of which is bound to inorganic polyphosphate while a smaller pool is subject to further transport (9, 10). During osmotic shock, Ca^2+^ is released from the vacuole through the transient receptor potential (TRP) channel ortholog Yvc1, leading to vacuole fission/fragmentation (11). This activity requires the phosphatidylinositol 3-phosphate (PI3P) 5-kinase Fab1 and the production of PI(3,5)P_2_, which is also linked to vacuole size and fragmentation (12, 13). Under isotonic conditions, Ca^2+^ efflux occurs during vacuole fusion upon the formation of *trans*-SNARE complexes through a Yvc1-independent mechanism (14). Unlike the fission pathway, vacuole fusion is inhibited by the overproduction of PI(3,5)P_2_ that is linked to the inhibition of net Ca^2+^ efflux (15, 16). PI(3,5)P_2_ lowers the observed net Ca^2+^ efflux through its activity on Pmc1. Taken together, it is likely that Fab1 activity serves as a switch that promotes fission while inhibiting fusion through its effects on Ca^2+^ transport.

The regulatory functions of Fab1 activity are not limited to Ca^2+^ transport and the fission/fusion switch. PI(3,5)P_2_ also affects V-ATPase activity through direct physical interactions with the V_O_ subunit Vph1 (17, 18). Vph1 is also found in a complex with Pmc1 and the R-SNARE Nyv1 that is sensitive to PI(3,5)P_2_ concentrations (16). The interaction between Vph1 and PI(3,5)P_2_ stabilizes the assembly of V_1_-Vo holocomplex to form the active V-ATPase. In the Golgi, Stv1 takes the place of Vph1 and interacts with the compartment rich lipid PI4P instead of PI(3,5)P_2_, which is only made on late endosomes and lysosomes (19). In both instances, specific phosphoinositides are essential for V-ATPase function.

The effects of PI(3,5)P_2_ on both V-ATPase function and Ca^2+^ transport suggest that these transport mechanisms could be interdependent. This notion is consistent with previous reports showing that inhibiting V-ATPase activity blocks the ability of Vcx1 to detoxify the cytoplasm after an increase in Ca^2+^ (20). Others have shown that the uptake of 500 μM ^45^Ca^2+^ is sensitive to ionophores such as CCCP and Nigericin (21).

In this study, we examined the role of PI(3,5)P_2_ on H^+^ transport and how H^+^ and Ca^2+^ transport affect each other. We demonstrate that PI(3,5)P_2_ overproduction augments vacuole acidification while the lack of the lipid reduces acidification. We also show that increasing extraluminal Ca^2+^ concentrations blocked vacuole acidification in a manner linked to Vcx1, while chelating Ca^2+^ accelerated acidification. Finally, we show that a fully functioning V-ATPase is needed for Ca^2+^ efflux. Together, this study shows that the transport of H^+^ and Ca^2+^ is interdependent and can be affected by the PI(3,5)P_2_ composition of the membrane.

## Results

### Proton influx is modulated by Fab1 activity

Others have shown that adding exogenous short chain dioctanoyl (C8) PI(3,5)P_2_ to purified yeast vacuoles augmented acidification (17). This was proposed to be due in part to the ability of PI(3,5)P_2_ to stabilize the V_1_–V_O_ holocomplex, through the interactions of the V_O_ subunit Vph1 and this lipid (18). However, the effect of adding C8-PI(3,5)P_2_ on stabilizing the V_1_–V_O_ complex has not been shown directly. Our previous work showed that elevating concentrations of PI(3,5)P_2_ inhibited vacuole fusion (15). This was later linked to the ability of PI(3,5)P_2_ levels to affect Ca^2+^ transport across the vacuolar membrane (16). Based on these findings, we hypothesized that PI(3,5)P_2_ may affect V-ATPase activity through modulating Ca^2+^ efflux from the vacuole lumen.

**Figure 1:**
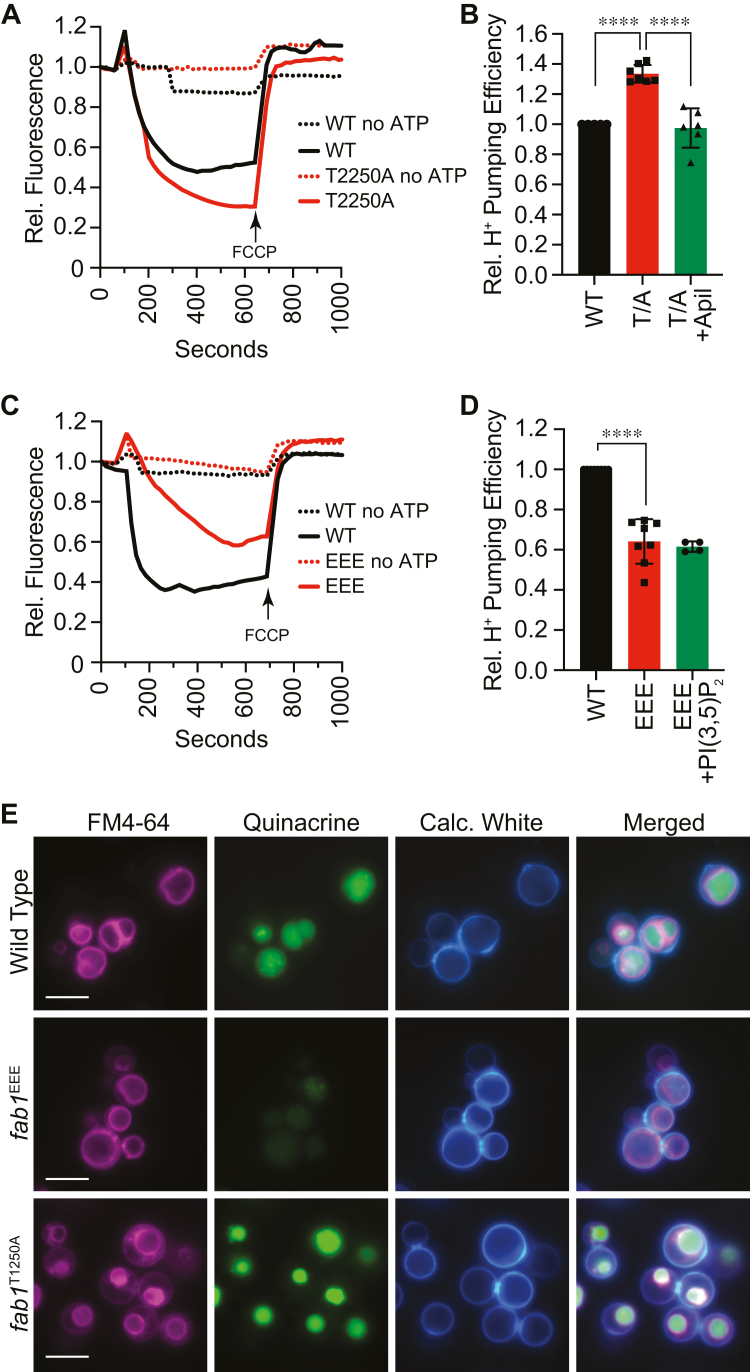
**Modifying PI(3,5)P**_**2**_**levels alters vacuole acidification.** Vacuoles were used for vacuole acidification measured by AO fluorescence. Reactions were incubated with or without ATP-regenerating system added at 30 s and incubated for a total of 600 s, at which point FCCP was added to equilibrate the H^+^ gradient. AO fluorescence was normalized to the initial value set to 1. *A*, WT and *fab1*^*T2250A*^ vacuoles were compared in their efficiency to acidify vacuoles. In parallel, *fab1*^*T2250A*^ vacuoles were incubated in the presence of 125 μM apilimod. *B*, quantitation of multiple experiments in panel (*A*) showed a significant effect of expressing *fab1*^*T2250A*^ compared to WT [F(2,15) = 36; ∗∗∗∗*p* < 0.0001] (One way ANOVA for multiple comparisons). Error bars are mean ± SD. Tukey’s multiple comparison test was used for individual *p* values. (n ≥ 4). *C*, WT and *fab1*^*EEE*^ vacuoles were compared in their acidification efficiency. Separately, *fab1*^*EEE*^ vacuoles were incubated in C8-PI(3,5)P_2_. *D*, quantitation of multiple experiments in panel (*C*) showed a significant effect of expressing *fab1*^*EEE*^ compared to WT [F(2,17) = 62.05; ∗∗∗∗*p* < 0.0001] (One way ANOVA for multiple comparisons). Error bars are mean ± SD. Tukey’s multiple comparison test was used for individual *p* values (n ≥ 4). *E*, WT, *fab1*^*T2250A*^, and *fab1*^*EEE*^ cells were stained with quinacrine to label acidified compartments. FM4-64 was used to stain the vacuole membrane and Calcofluor (Calc.) White was used to stain the cell wall. The scale bar represents 5 μm. AO, acridine orange; FCCP, carbonyl cyanide-4-(trifluoromethoxy) phenylhydrazone.

To test our hypothesis, we started by recapitulating the published results using C8-PI(3,5)P_2_ and other C8 lipids and found that C8-PI(3,5)P_2_ indeed enhanced acidification as measured by changes in acridine orange (AO) fluorescence; however, the changes were modest in our hands (not shown). This prompted us to ask whether changing endogenous production of the lipid would have a stronger effect. To do this, we performed AO fluorescence assays with vacuoles isolated from WT yeast as well as strains expressing the kinase-deficient *fab1*^EEE^ or the hyperactive *fab1*^T2250A^ mutations (18, 22). AO fluorescence is reduced at 520 nm in acidic environments while increasing in fluorescence a 680 nm (23, 24). Thus, the red-spectral shift in AO fluorescence serves as a measure of vacuole acidification. Vacuoles were incubated with AO for 600 s to allow acidification to plateau. Carbonyl cyanide-4-(trifluoromethoxy) phenylhydrazone (FCCP) was added after 600 s to collapse the proton gradient and show that the change in AO fluorescence was due to active ATP-dependent transport and not passive transport of the dye (25). In Figure 1, *A* and *B*, we show that the overproduction of PI(3,5)P_2_ by *fab1*^T2250A^ led to a pronounced increase in acidification, as manifested by an augmented drop in AO fluorescence. To show that the increased H^+^ transport activity by *fab1*^T2250A^ vacuoles was indeed due to the overproduction of PI(3,5)P_2_, we added the PIKfyve/Fab1 inhibitor apilimod to reactions containing *fab1*^T2250A^ vacuoles (16, 26, 27). We found that apilimod restored *fab1*^T2250A^ vacuole acidification to WT levels (Fig. 1*B*). Previously, we showed elevated levels of PI(3,5)P_2_, whether added exogenously as C8-PI(3,5)P_2_ or overproduce by *fab1*^T2250A^ led to prolonged Ca^2+^ uptake by Pmc1 (16). Importantly, we found that adding apilimod reversed the effect of overproducing PI(3,5)P_2_ by *fab1*^T2250A^ to WT levels. Together with the current data, it suggests that PI(3,5)P_2_ may link Ca^2+^ and H^+^ transport on the yeast vacuole.

While the increase in PI(3,5)P_2_ led to enhanced acidification, we next asked if the lack of PI(3,5)P_2_ would prevent the spectral shift AO fluorescence. To test this, we used vacuoles harboring the kinase-deficient *fab1*^EEE^ mutant (28). This showed that *fab1*^EEE^ vacuoles had attenuated vacuole acidification (Fig. 1, *C* and *D*), which is in keeping with the destabilization of the V_1_–V_O_ complex when PI(3,5)P_2_ is absent (22). That said, enough V_1_–V_O_ remained on the vacuole to partially acidify the vacuole lumen. To verify if the difference was due to the absence of PI(3,5)P_2_, we supplemented the reaction with C8-PI(3,5)P_2_. Curiously, our data showed that supplementing *fab1*^EEE^ vacuoles with C8-PI(3,5)P_2_ did not restore acidification (Fig. 1*D*). In contrast, adding C8-PI(3,5)P_2_ to WT vacuoles did augment acidification as shown by the Kane lab (17). The inability to rescue acidification does not mean that the lipid has no effect on *fab1*^EEE^ vacuoles. In other studies, we have shown that C8-PI(3,5)P_2_ restored Ca^2+^ transport by *fab1*^EEE^ vacuoles to WT levels (16). The lack of an effect could be multifactorial. The simplest answer is that C8-PI(3,5)P_2_ restored Ca^2+^ transport by *fab1*^EEE^ vacuoles due to reestablishing on-site interactions of factors present after vacuole isolation, whereas the lack of an effect on AO fluorescence could reflect the absence of a factor that required PI(3,5)P_2_ *in vivo* prior to vacuole isolation. For instance, we have shown that *fab1*^EEE^ vacuoles have diminished levels of the ABC transporter Ycf1, which interacts with the V_1_ subunit Vma10 (15, 29). While this interaction has not been tested for its effects on V-ATPase efficiency, it serves to illustrate that a more complex network of interactions could be linked to the observed phenotype. In addition, mass spectrometry analysis of *fab1*^EEE^ vacuoles showed that they contained more of the polyphosphate synthase Vtc4 (not shown) (30). Deletion of *VTC4* inhibits V-ATPase activity; however, overproduction has not been tested on vacuole acidification. Finally, others have shown that PI(3,5)P_2_ can activate TORC1 and its phosphorylation of the kinase Sch9, which in turn affects the assembly and function of V-ATPases (31, 32, 33, 34). Thus, it is possible that *fab1*^EEE^ vacuoles have faulty Sch9 function that can alter vacuole acidification in a way that cannot be restored *in vitro* by the addition of C8-PI(3,5)P_2_. Nevertheless, it is evident that modulating endogenous PI(3,5)P_2_ production can augment or dampen vacuole acidification. The lack of a complete inhibition of proton pumping by *fab1*^EEE^ vacuoles is in accord with a study showing that *fab1*Δ vacuoles were able to acidify as measured with cDCFDA [5-(and-6)-carboxy-2′,7′-dichlorofluorescein diacetate], an esterase dye that is pH sensitive, and pHluorin fluorescence (35). On the other hand, the original work looking at *fab1*Δ found that the deletion lead to a rise in vacuolar pH from 6 to 7 (36). Still, others have found that phagosomes that lack Fab1 are not acidified, leading to reduced proteolysis, immature Cathepsin D, and inhibited bacterial killing (37).

To confirm the effects of expressing *fab1* mutants on vacuole acidification, we looked at whole cells stained with FM4-64 to label the vacuole and quinacrine to label acidified compartments. Calcofluor White was used to stain the cell walls. As shown in Figure 1*E*, the vacuoles of WT cells stained brightly with quinacrine. In comparison, cells expressing *fab1*^EEE^ only lightly stained with quinacrine, further indicating that cells lacking PI(3,5)P_2_ are defective in vacuole acidification. The reduction in quinacrine fluorescence in *fab1*^EEE^ relative to WT was starker that what was seen with AO fluorescence, suggesting that tracking AO fluorescence using a plate reader was more sensitive compared to quinacrine and fluorescence microscopy. Finally, we found that *fab1*^T2250A^ vacuoles stained more brightly compared to WT, which was indicative of augmented acidification and agreed with what was seen with AO fluorescence. While examining *fab1*^EEE^ is informative, the remainder of the study focuses on the overproduction of PI(3,5)P_2_ by *fab1*^T2250A^.

### Sequestering PI(3,5)P_2_ affects vacuole acidification

**Figure 2:**
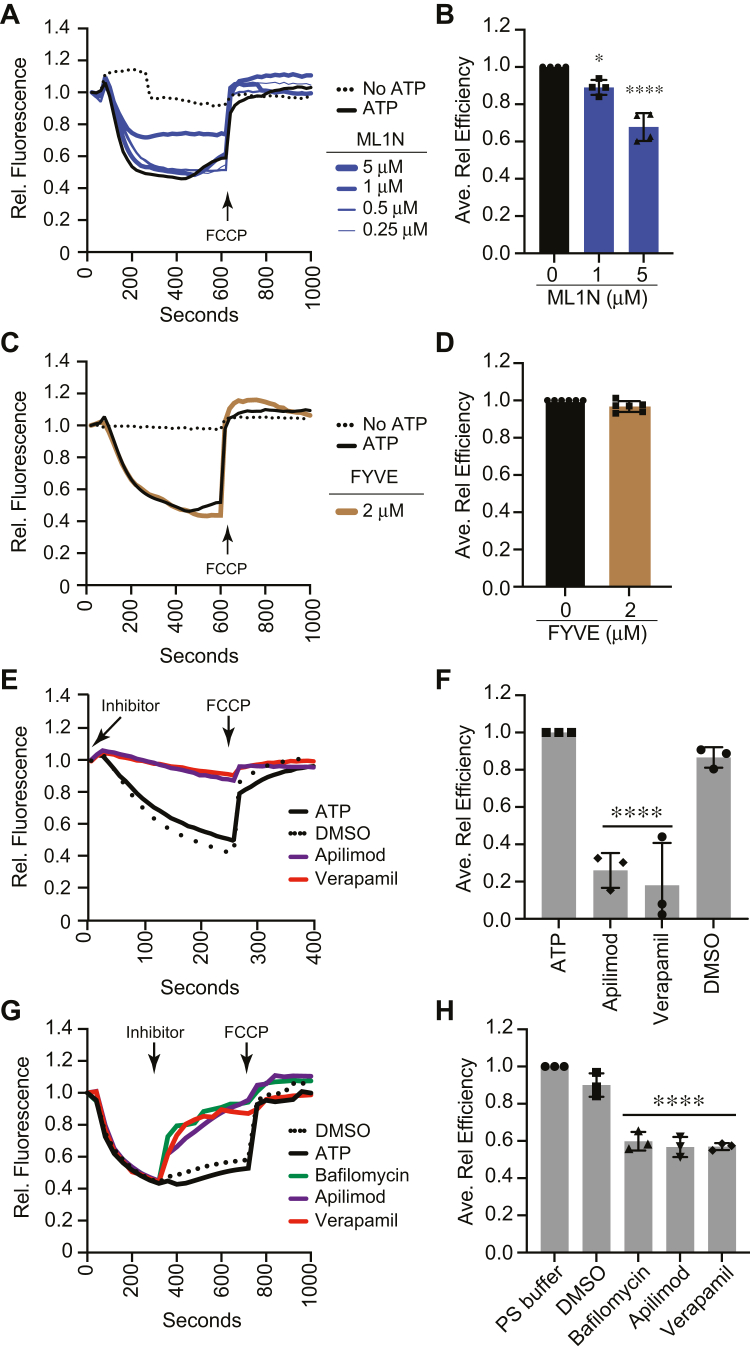
**Sequestering or producing PI(3,5)P**_**2**_**inhibits vacuole acidification.***A*, WT vacuoles were incubated with a dose curve of the PI(3,5)P_2_ binding domain GST-ML1N and AO fluorescence was measured. *B*, quantitation of multiple experiments in panel (*A*) showed a significant effect of treating reactions with ML1N [F(2,9) = 44.48; ∗∗∗∗*p* < 0.0001] (One way ANOVA for multiple comparisons with no treatment (0 μM) as a control). Error bars are mean ± SD. Dunnett multiple comparison test was used for individual *p* values (n = 4). ∗*p* < 0.05, ∗∗∗∗*p* < 0.001. *C*, WT type vacuoles were incubated with GST-FYVE and AO quenching was measured. *D*, average of multiple experiments in panel (*C*). *E*, vacuoles were treated with 125 μM apilimod, 250 μM verapamil, or DMSO (solvent) in the presence of AO at the beginning of the reaction. AO fluorescence was measured for 250 s and FCCP was added seconds to collapse the H^+^ gradient (*arrow*). *F*, quantitation of multiple experiments in panel (*E*) showed a significant effect of treating reactions with apilimod and verapamil [F(6,14) = 56.02; ∗∗∗∗*p* < 0.0001] (One way ANOVA for multiple comparisons with no treatment (0 μM) as a control). Error bars are mean ± SD. Dunnett multiple comparison test was used for individual *p* values (n = 3). ∗∗∗∗*p* < 0.001. *G*, effect of late additions of 125 μM apilimod, 250 μM verapamil, 100 nM bafilomycin, and DMSO on H^+^ transport (*arrow*, Inhibitor) added at 350 s. FCCP was added at 720 s to collapse the H^+^ gradient (*arrow*, FCCP). *H*, quantitation of multiple experiments in panel (*G*) showed a significant effect of treating reactions with bafilomycin, apilimod, and verapamil [F(4,10) = 65.72; ∗∗∗∗*p* < 0.0001] (One way ANOVA for multiple comparisons with no treatment (Late PS) as a control). Error bars are mean ± SD. Dunnett multiple comparison test was used for individual *p* values (n = 3). ∗∗∗∗*p* < 0.001. AO, acridine orange; DMSO, dimethyl sulfoxide; FCCP, carbonyl cyanide-4-(trifluoromethoxy) phenylhydrazone.

In Figure 1, we showed that *fab1* mutations affected vacuole acidification; however, it was unclear whether PI(3,5)P_2_ was directly involved. To test this, we sequestered PI(3,5)P_2_ with a specific lipid binding protein. We used the N terminus (ML1N) of the endolysosomal TRP Mucolipin-1/TRPML1 Ca^2+^ channel (11). ML1N binds to vacuolar PI(3,5)P_2_ and inhibits vacuole fusion (15). Here, we found GST-ML1N reduced the spectral shift of AO fluorescence, indicating that vacuole acidification was blocked by sequestering PI(3,5)P_2_ (Fig. 2, *A* and *B*). As a control, we used FYVE domain to bind PI3P at concentrations that inhibit vacuole fusion (38, 39, 40). Adding FYVE domain had no effect on AO fluorescence, showing binding PI3P does not alter vacuole acidification (Fig. 2, *C* and *D*). We have also used the PI4P binding PH domain from FAPP1 at concentrations that inhibit vacuole fusion and saw no effect on acidification (41, 42, 43, 44). This underscores the importance of free PI(3,5)P_2_ in vacuole acidification.

### Fab1 activity links vacuole acidification to Ca^2+^ transport

In the results aforementioned, we used Fab1 mutations to examine the effects of PI(3,5)P_2_ production during AO fluorescence experiments. To test if inhibiting Fab1 on WT vacuoles would have similar effects, we used the Fab1 inhibitor apilimod. We have previously shown that apilimod inhibits PI(3,5)P_2_ production by Fab1 on isolated vacuoles (16). Here, we found that adding apilimod at the start of the reaction completely blocked the spectral shift in AO fluorescence, suggesting that inhibiting PI(3,5)P_2_ production had inhibited vacuole acidification (Fig. 2, *E* and *F*). The dimethyl sulfoxide (DMSO) treatment had no effect, showing that the effect of apilimod was not due to its solvent.

Due to the effects of apilimod, we asked if Ca^2+^ transport directly affected vacuole acidification. Isolated vacuoles rapidly take up Ca^2+^ from the medium upon adding ATP, after which they release Ca^2+^ during the docking stage of fusion in a SNARE-dependent manner (14). The take up of Ca^2+^ can be inhibited by compounds that target Ca-ATPase pumps. In a previous study, we found that the Ca^2+^ pump inhibitor verapamil blocked Ca^2+^ uptake when added at the start of the assay, suggesting that the sole vacuolar Ca-ATPase pump Pmc1 was inhibited (16). This indicated that the Pmc1 is the primary mechanism for Ca^2+^ uptake since the Ca^2+^/H^+^ antiporter does not use ATP. This was further supported by inhibition of Ca^2+^ uptake by verapamil on *vcx1*Δ vacuoles. Vacuole acidification was also blocked by the Ca-ATPase inhibitors cyclopiazonic acid and prenylamine (45, 46, 47) (not shown).

Knowing the effects of verapamil on Ca^2+^, we asked if vacuole acidification would be affected in a similar manner. When we added verapamil to AO fluorescence assays at the start of the experiment, we observed a complete block in acidification (Fig. 2, *E* and *F*). Because these treatments also inhibited the uptake of Ca^2+^, it suggested that maintaining elevated extraluminal Ca^2+^ could inhibit vacuole acidification (16). It further suggests that the effects of PI(3,5)P_2_ on vacuole acidification could be linked to modulating Ca^2+^ transport.

### Inhibiting PI(3,5)P_2_ production and blocking Ca^2+^ transport disrupts vacuolar H^+^ gradients

Previously, we found that apilimod added after 10 min of Ca^2+^ uptake led to a halt in uptake followed by an efflux of Ca^2+^ (16). This indicated that Fab1 continued to be active and that PI(3,5)P_2_ was required for normal Ca^2+^ transport across the vacuole membrane. Here, we asked whether Fab1 activity was also required for maintaining an acidified lumen. To test this, we added apilimod after 300 s when AO quenching had plateaued upon forming a stable H^+^ gradient of acidification. We found that AO fluorescence at 520 nm rapidly increased upon adding apilimod, indicating that vacuole acidification was no longer maintained (Fig. 2, *G* and *H*). Adding DMSO had no effect on vacuole acidification, indicating that the effect was due to apilimod. Similarly, adding verapamil at 300 s led to a rapid deacidification. Prenylamine has similar effect (not shown). The loss of the H^+^ gradient was consistent with the rapid release of Ca^2+^ when vacuoles were treated with verapamil after 600 s of incubation (16). The effects of apilimod and verapamil were not due to leakage/lysis as these reagents do not inhibit vacuoles from fusing (16). Verapamil and others inhibitor of Ca^2+^ transport including nicardipine, terodiline, and diltiazem also block the formation of H^+^ gradients produced by V-ATPase activity in catecholamine storage vesicles, indicating that a Ca^2+^ gradient was needed for vesicle acidification (48). Together, these data bolster the idea that PI(3,5)P_2_, Ca^2+^ transport, and vacuole acidification are part of a regulatory circuit. As a control, we used bafilomycin to block V-ATPase activity. This led to the deacidification of the vacuoles, indicating that the H+ gradient must be constantly maintained through V-ATPase activity.

### Ca^2+^ affects V-ATPase activity

Based on the effects of apilimod and verapamil on vacuole acidification and Ca^2+^ transport, we asked whether the direct addition or sequestration of Ca^2+^ would affect vacuole acidification. Others have shown that adding Ca^2+^ at millimolar levels inhibited the fusion WT vacuoles while *vcx1*Δ/*pmc1*Δ or *vcx1*Δ vacuoles were resistant (49). That study also showed that while WT vacuoles accumulated quinacrine at a reduced rate in the presence of Ca^2+^, the *vcx1*Δ/*pmc1*Δ vacuoles took up quinacrine at untreated WT levels. The resistance to Ca^2+^ was attributed to Vcx1 function and suggests that high Ca^2+^ levels need to enter vacuoles to prevent fusion and acidification. Their experiments used quinacrine to measure acidification in an endpoint assay. This approach misses the dynamics of acidification and could miss changes in kinetics even if the endpoint is the same. As shown previously, quinacrine is likely to miss small changes in acidification.

**Figure 3:**
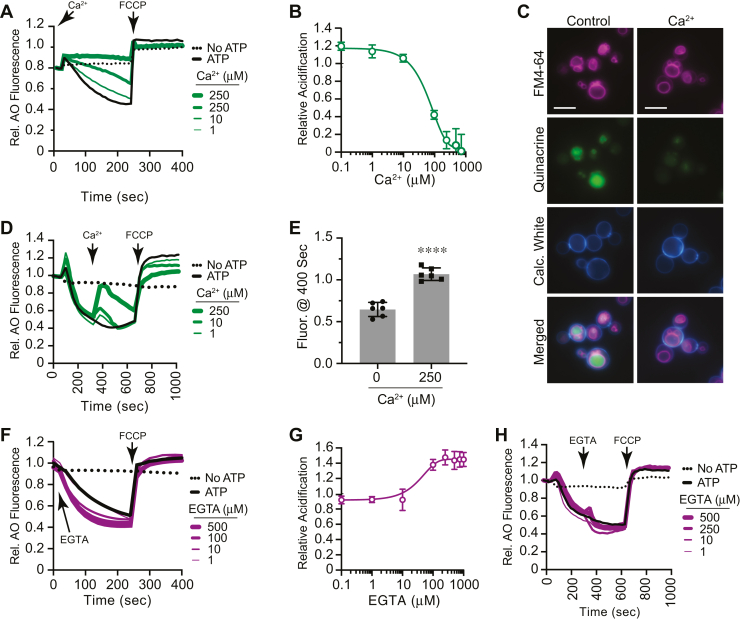
**Ca**^**2+**^**level modulation alters acidification.***A*, AO fluorescence assays were performed in the presence of a dosage curve of CaCl_2_ at the indicated concentrations or PS buffer added at the start of the reactions. A separate reaction omitted ATP. Reactions were incubated for 240 s, after which FCCP was added (*arrow*). AO fluorescence was normalized to the initial value set to 1. *B*, average of multiple experiments showing the effects of Ca^2+^ on AO fluorescence. *C*, log-phase BJ3505 cells were incubated with 200 μM quinacrine and 2 μM FM4-64 for with or without CaCl_2_ for 1 h. Cell walls were stained with Calcofluor White. The scale bar represents 5 μm. *D*, vacuoles were incubated for 300 s at which point a curve of Ca^2+^ was added and further incubated for a total of 700 s before addition of FCCP. *E*, average of AO fluorescence when treated with 250 μM Ca^2+^ or buffer alone. Error bars represent mean ± SD. (n = 6). Data was analyzed using a Student’s unpaired two-tailed *t* test ∗∗∗∗*p* < 0.0001. *F*, vacuole acidification in the presence of a dosage curve of EGTA or PS buffer. *G*, average of multiple experiments showing the effect EGTA on acidification. *H*, AO fluorescence reactions were incubated for 300 s, after which a concentration curve EGTA was added and further incubated for a total of 700 s before adding FCCP. AO, acridine orange; FCCP, carbonyl cyanide-4-(trifluoromethoxy) phenylhydrazone.

To test the connection between PI(3,5)P_2_, Ca^2+^, and vacuole acidification, we needed to see how Ca^2+^ affected AO fluorescence. First, we performed AO fluorescence assays with isolated vacuoles incubated with a dosage curve of CaCl_2_. This showed that Ca^2+^ inhibited vacuole acidification in a dose-dependent manner at micromolar levels (Fig. 3, *A* and *B*). Full inhibition of acidification occurred with 250 μM Ca^2+^, which was nearly 10-fold less that what was has been shown to inhibit fusion (49). To verify the effects of Ca^2+^ on vacuole acidification, we used fluorescence microscopy and quinacrine staining of whole cells (50, 51). As expected, untreated cells accumulated quinacrine in their vacuoles and fluoresced strongly (Fig. 3*C*). When cells were incubated with CaCl_2_, we observed that quinacrine staining was blocked. This is in accord with the AO fluorescence data in Figure 3, *A* and *B*.

After determining that adding excess Ca^2+^ inhibited vacuole acidification, we also tested if it affected vacuole acidification after the H^+^ gradient has been established. To answer this, we added a dosage curve of Ca^2+^ after 300 s of incubation, which is enough time to acidify vacuoles. This showed a dose-dependent deacidification of vacuoles as shown by an increase in AO fluorescence at 520 nm (Fig. 3, *D* and *E*). This suggests one of two possibilities. First, it could be that the addition of Ca^2+^ at this point in the assay uses the antiporter activity of Vcx1, which would expel H^+^ as it takes up the newly added Ca^2+^. Alternatively, it could indicate that the added Ca^2+^ altered V-ATPase efficiency, resulting in the accumulation of extraluminal H^+^ (Fig. 3*E*). This was similar to findings by Cagnac *et al.*; however, they added bafilomycin A1 before adding Ca^2+^, preventing a direct comparison (52). It should be noted that AO fluorescence was restored when 250 μM Ca^2+^ was added late in the reaction (Fig. 3*E*), while addition at the beginning prevented the fluorescence shift of AO for the duration of the experiment (Fig. 3*A*). This suggests that once vacuoles have established a H^+^ gradient equilibrium, they are able to quickly recover from the Ca^2+^ spike and reestablish the H^+^ gradient.

The role of a Ca^2+^ gradient in vacuole acidification could also be tested by chelating extraluminal Ca^2+^ with EGTA. This would make a near instant gradient of free lumenal Ca^2+^ without the need of Pmc1 or Vcx1 function and accelerate vacuole acidification. As hypothesized, we found that EGTA enhanced the rate of acidification, suggesting that Ca^2+^ uptake precedes acidification (Fig. 3, *F* and *G*). Based on the effects of adding Ca^2+^ late in the reaction, we asked if adding EGTA would further enhance H^+^ uptake when added late. Unlike the effects of excess Ca^2+^, the addition of EGTA at 300 s did not affect acidification (Fig. 3*H*). We attribute this to the relative absence of extraluminal Ca^2+^ after 300 s of incubation as Ca^2+^ uptake is typically completed between 300 and 500 s (15, 16, 53, 54). Together, these data indicate that vacuole acidification was affected by changes in the vacuolar Ca^2+^ gradient. This was also consistent with the notion that Fab1 activity correlates with vesicle acidification through modulating extraluminal Ca^2+^ levels.

### Vacuole acidification in Fab1 mutants is inhibited by high levels of Ca^2+^

**Figure 4:**
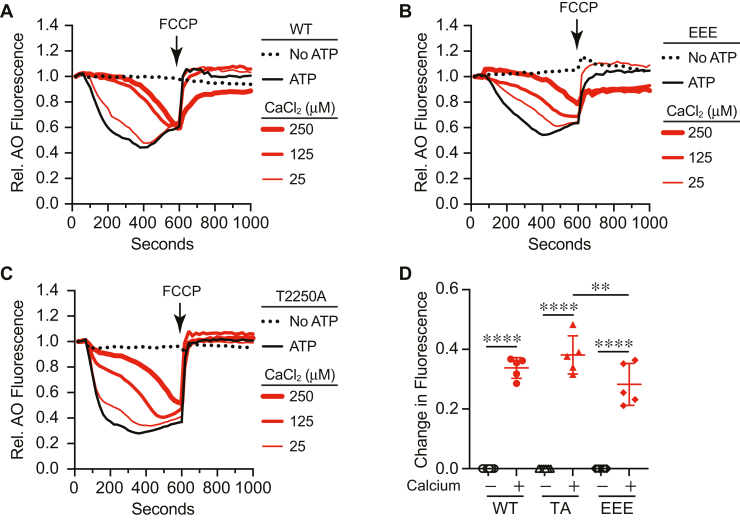
**Effect of Ca**^**2+**^**on *fab1* mutants.** Vacuoles from WT (*A*), *fab1*^EEE^ (*B*), and *fab1*^T2250A^ (*C*) were incubated with buffer or increasing levels of Ca^2+^ for 600 s. Separate reactions were performed in the absence of ATP. FCCP was added to all reactions at 600 s to collapse the H^+^ gradient. AO quenching was normalized to the initial fluorescence for each reaction that was set to 1. *D*, quantitation of multiple experiments showed a significant effect of treating reactions with 125 μM Ca^2+^ [F(5,28) = 130.9; ∗∗∗∗*p* < 0.0001] (One way ANOVA for multiple comparisons). Error bars are mean ± SD. Tukey’s multiple comparison test was used for individual *p* values (n = 5). ∗∗*p* < 0.01, ∗∗∗∗*p* < 0.001, ns, not significant. AO, acridine orange; FCCP, carbonyl cyanide-4-(trifluoromethoxy) phenylhydrazone.

In a previous study, we showed that Ca^2+^ uptake was prolonged in the presence of the *fab1*^T2250A^ hyperactive mutant while Ca^2+^ uptake was attenuated when the *fab1*^EEE^ inactive mutant was expressed (16). Based on this, we predicted that *fab1*^T2250A^ vacuoles would be resistant to added Ca^2+^ relative to the WT and that *fab1*^EEE^ vacuoles would be more sensitive. Instead, we found that WT, *fab1*^T2250A^, and *fab1*^EEE^ vacuoles were equally sensitive to elevated Ca^2+^ at the micromolar levels needed to block vacuole acidification *in vitro* (Fig. 4). While this suggests that Fab1 activity does not affect acidification through Ca^2+^ flux, it is possible that the effects of high micromolar Ca^2+^ overwhelm the system causing it to act as if under shock conditions. If this is so, then the relationship between Ca^2+^, PI(3,5)P_2_, and vacuole acidification can be split into two major tracks. The first track occurs under homeostatic conditions where Ca^2+^ is at normal nontoxic levels. Here, Ca^2+^ uptake into the vacuole is primarily through the high affinity pump Pmc1 (6). Under these conditions, Pmc1 activity is linked to vacuole acidification and Fab1 activity. This notion is supported by the interactions between Pmc1 and Vph1 that is sensitive to C8-PI(3,5)P_2_, albeit at moderate levels (16). The second track is taken when Ca^2+^ is at toxic levels and the low affinity antiporter Vcx1 takes up Ca^2+^ while deacidifying the vacuole through H^+^ expulsion (8). The second track would bypass the need for the interactions between Pmc1, Vph1, and PI(3,5)P_2_. Thus, it is important to directly compare how vacuole lacking Vcx1 or Pmc1 reacts to added Ca^2+^.

### High Ca^2+^ levels alter vacuole acidification through Vcx1

To determine which vacuolar Ca^2+^ transporter was linked to the effects on acidification activity, we used a panel of deletion strains lacking the Ca^2+^ exporter channel Yvc1, the Ca^2+^-ATPase importer pump Pmc1, and the Ca^2+^/H^+^ exchanger Vcx1. Yeast vacuoles are thought to only take up Ca^2+^ through Pmc1 and Vcx1, therefore, the deletion of one can function as a reporter for the other. Previous work by Ungermann *et al.* indicated that the inhibition of vacuole fusion by millimolar Ca^2+^ occurred through Vcx1 (49). In the absence of Vcx1, they showed that vacuole fusion was resistant to Ca^2+^ and able to take up quinacrine. The quinacrine assays were endpoint experiments using a single concentration of Ca^2+^ at 1.5 mM. Thus, it is possible that using lower micromolar levels of Ca^2+^ in a real-time assay could detect changes that were previously missed.

**Figure 5:**
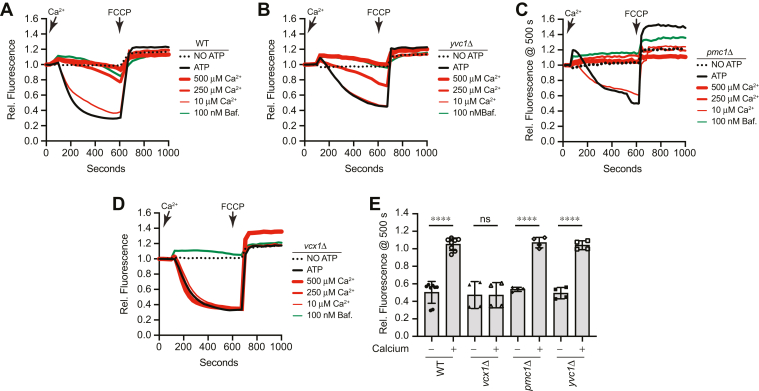
**Effect of Ca**^**2+**^**on vacuole acidification with Ca**^**2+**^**transporter mutants.** Vacuoles from WT (*A*), *yvc1*Δ (*B*), *pmc1*Δ (*C*), and *vcx1*Δ (*D*) yeast strains were incubated with buffer, bafilomycin A1, or a curve a CaCl_2_ for 600 s. A separate reaction was performed in the absence of ATP. After ∼600 s, 30 μM FCCP was added to collapse the H^+^ gradient. AO fluorescence was normalized to the initial values set to 1. *E*, average changes in fluorescence in the absence or presence of 125 μM CaCl_2_ [F(7,32) = 44.27; ∗∗∗∗*p* < 0.0001] (One way ANOVA for multiple comparisons). Error bars are mean ± SD. Tukey’s multiple comparison test was used for individual *p* values (n ≥ 4). ∗∗∗∗*p* < 0.001. AO, acridine orange; FCCP, carbonyl cyanide-4-(trifluoromethoxy) phenylhydrazone.

Here, WT and Ca^2+^ transporter mutant vacuoles were used in AO fluorescence assays in the presence of a CaCl_2_ concentration curve. We found that the shift in AO fluorescence was inhibited when WT vacuoles were incubated in the presence of ≥250 μM Ca^2+^ as seen previously, showing again that vacuole acidification was blocked (Fig. 5, *A* and *E*). Similarly, the shift in AO fluorescence using vacuoles from *yvc1*Δ and *pmc1*Δ cells was inhibited by ≥250 μM Ca^2+^ (Fig. 5, *B*, *C* and *E*). The results with *yvc1*Δ vacuoles were as predicted since the TRP channel is an exporter of luminal Ca^2+^. The sensitivity seen with *pmc1*Δ vacuoles suggests that Vcx1 mediated transport of Ca^2+^ is linked to vacuole deacidification. When *vcx1*Δ vacuoles were tested, it showed that they were indeed resistant to the Ca^2+^ all concentrations (Fig. 5, *D* and *E*). Together, these data suggest that the excess added Ca^2+^ is taken up through Vcx1, leading to the expulsion of H^+^ through its antiporter activity. This is also in agreement with previous studies.

**Figure 6:**
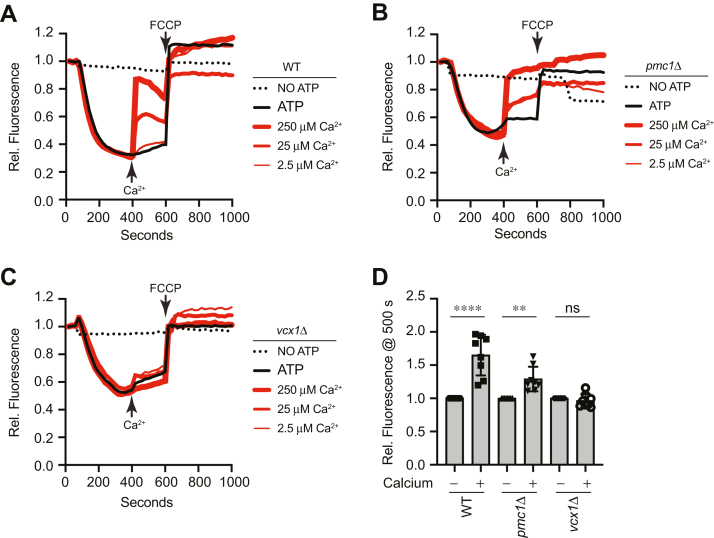
**Late addition of Ca**^**2+**^**and vacuole acidification.** Vacuoles from WT (*A*), *pmc1*Δ (*B*), and *vcx1*Δ (*C*) yeast strains were incubated with buffer for 400 s. A separate reaction was performed in the absence of ATP. At 400 s, individual reactions were supplemented with additional reaction buffer or CaCl_2_ at the indicated concentrations. After ∼600 s, 30 μM FCCP was added to collapse the H^+^ gradient. AO fluorescence was normalized to the initial values set to 1. *D*, quantitation of multiple experiments in panel (*C*) showed a significant effect of treating reactions with Ca^2+^ after 600 s [F(5,42) = 25.85; ∗∗∗∗*p* < 0.0001] (One way ANOVA for multiple comparisons). Error bars are mean ± SD. Tukey’s multiple comparison test was used for individual *p* values (n ≥ 4). ∗∗*p* < 0.01, ∗∗∗∗*p* < 0.001. AO, acridine orange; FCCP, carbonyl cyanide-4-(trifluoromethoxy) phenylhydrazone.

We next examined whether the effects of Ca^2+^ on V-ATPase activity also occurred after vacuoles had acidified. We added Ca^2+^ after 400 s of incubation and continued measuring fluorescence for 200 additional seconds before the addition of FCCP. WT and *pmc1*Δ vacuoles showed that the late addition of Ca^2+^ resulted in a dose-dependent increase in AO fluorescence at 520 nm, illustrating that H^+^ was released (Fig. 6, *A*, *B* and *D*). This was comparable to what was seen with the late addition of bafilomycin. When *vcx1*Δ vacuoles were tested, we again saw a resistance toward the added Ca^2+^ (Fig. 6, *C* and *D*). This further indicates that vacuole deacidification in the presence of micromolar levels of Ca^2+^ was linked to Vcx1 antiporter activity that released H^+^ as the result of taking in Ca^2+^.

### Cadmium and zinc do not inhibit vacuole acidification

Others have shown that the addition of Ca^2+^ to synaptic vesicles led to the release of H^+^ with varying degrees of intensity (55, 56, 57). Using synaptic vesicles investigators have also show that the addition of Zn^2+^ and Cd^2+^ triggered an equivalent or more potent release of H^+^ *versus* adding Ca^2+^ (56, 57). In rat kidney vesicles from brush border membranes, the addition of Cd^2+^ inhibited V-ATPase activity (58). Zn^2+^ has also been shown to both inhibit and enhance V-ATPase function in different plants (59, 60, 61). Finally, Cagnac *et al.* showed that both Cd^2+^ and Zn^2+^ could affect yeast vacuole acidification in a manner linked to Ca^2+^/H^+^ antiporter activity (52). Together, these reports indicated that divalent cations other than Ca^2+^ can affect V-ATPase activity, albeit in different systems and with varying results.

**Figure 7:**
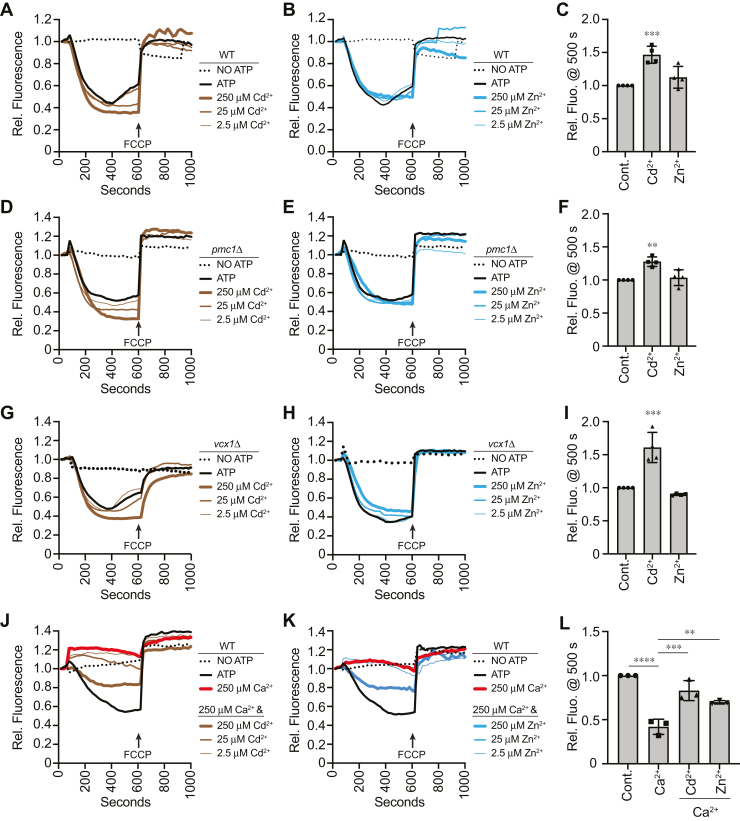
**Effect of Cd**^**2+**^**and Zn**^**2+**^**on vacuole acidification.** Vacuoles from WT (*A*–*C*), *pmc1*Δ (*D*–*F*), and *vcx1*Δ (*G*–*I*) yeast strains were incubated with buffer, CdCl_2_, or ZnCl_2_ at the indicated concentrations. Reactions were incubated for 600 s, after which FCCP was added to collapse the H^+^ gradient. Average peak fluorescence quenching normalized to the initial fluorescence set to 1. The untreated control was normalized to 1 and relative increases or decreases were calculated for Cd^2+^ and Zn^2+^ treatments. *J*–*L*, WT vacuoles were treated with a combination of 250 μM CaCl_2_ with concentration curves of Cd^2+^ (*J*) or Zn^2+^ (*K*). (*L*) average peak fluorescence quenching. Untreated control was set to 1 and relative changes in fluorescence were calculated for each treatment. Quantitation of multiple experiments showed a significant effect of treating reactions with Cd^2+^ and Zn^2+^. Data were analyzed using one-way ANOVA for multiple comparisons [F(2,9) = 16; ∗∗∗∗*p* = 0.0011] for (*C*), [F(2,9) = 13.99; ∗∗∗∗*p* = 0.0017] for (*F*), [F(2,9) = 33.79; ∗∗∗∗*p* < 0.0001] for (*I*), [F(3,8) = 34.94; ∗∗∗∗*p* < 0.0001] for (*L*). Error bars are mean ± SD. Tukey’s multiple comparison test was used for individual *p* values (n ≥ 3). ∗∗ *p* < 0.01, ∗∗∗ *p* < 0.001, ∗∗∗∗*p* < 0.0001. FCCP, carbonyl cyanide-4-(trifluoromethoxy) phenylhydrazone.

We used WT or Ca^2+^ transport deletion strains to test the effects Cd^2+^ and Zn^2+^. Contrary to what others found, we saw that Cd^2+^ addition significantly enhanced acidification in WT vacuoles (Fig. 7, *A* and *C*). Similar effects were observed with *pmc1*Δ vacuoles (Fig. 7, *D* and *F*) and *vcx1*Δ vacuoles (Fig. 7, *G* and *I*). When Zn^2+^ was tested, we found that there was no significant effect on any of the vacuole types (Fig. 7, *B*, *C*, *E*, *F*, *H* and *I*). Together, these data indicate that Cd^2+^ and Zn^2+^ do not reproduce the effects seen by the addition of Ca^2+^. Because of the activating effect of Cd^2+^ on vacuole acidification, we next asked if it could reverse the effects of Ca^2+^. To do this, we added a fixed amount of Ca^2+^ (250 μM) along with a curve of Cd^2+^. This showed that Cd^2+^ was able to partially restore vacuole acidification in the presence of Ca^2+^ (Fig. 7, *J* and *L*). Interestingly, Zn^2+^ had a similar effect on restoring the effects of Ca^2+^ on acidification, even though it had no effect on its own (Fig. 7, *K* and *L*). While the mechanism is unknown, the effect of Cd^2+^ could be attributed to its role as a mimetic and directly competing with Ca^2+^ for binding sites while not having an inhibitory effect. This is especially important to consider since Cd^2+^ can affect calmodulin (CaM)-dependent kinase II (CaMK-II) function and alter cytoskeletal dynamics as well as apoptosis (62). Less is known about the competition of Zn^2+^ and Ca^2+^; however, Zn^2+^ has been shown to alter Ca^2+^ binding to Calbindin D28K (63). Based on what we know now, we speculate that Cd^2+^ and Zn^2+^ compete with, or alter, the binding of Ca^2+^ to its site of action during V-ATPase activity.

### Effects of altering V-ATPase function on Ca^2+^ transport

Thus far, we have shown that modulating Ca^2+^ concentrations affected vacuole acidification. We next asked whether V-ATPase activity could in turn affect Ca^2+^ transport. The transport of Ca^2+^ in and out of the vacuole was detected by the Ca^2+^ binding fluorophore Cal-520 dextran conjugate, which fluoresces when bound to Ca^2+^ (15, 16). Upon the addition of ATP, vacuoles transport Ca^2+^ from the medium into the vesicle lumen, which is seen by the loss in fluorescence. In contrast, the omission of ATP prevents Ca^2+^ from entering the vacuole, and fluorescence remains mostly unchanged through the duration of the experiment. Ca^2+^ is later released when SNAREs form complexes between vacuoles (14). SNARE-dependent Ca^2+^ efflux is inhibited by various agents that block the fusion pathway prior *trans*-SNARE pairing including anti-Sec17 IgG, which stops the pathway at the priming stage (64, 65). Our previous work showed that increasing levels of PI(3,5)P_2_ blocked Ca^2+^ efflux and fusion (16). Interestingly, PI(3,5)P_2_ does not affect *trans*-SNARE pairing, suggesting it has its effects between *trans*-SNARE pairing and Ca^2+^ efflux (15).

**Figure 8:**
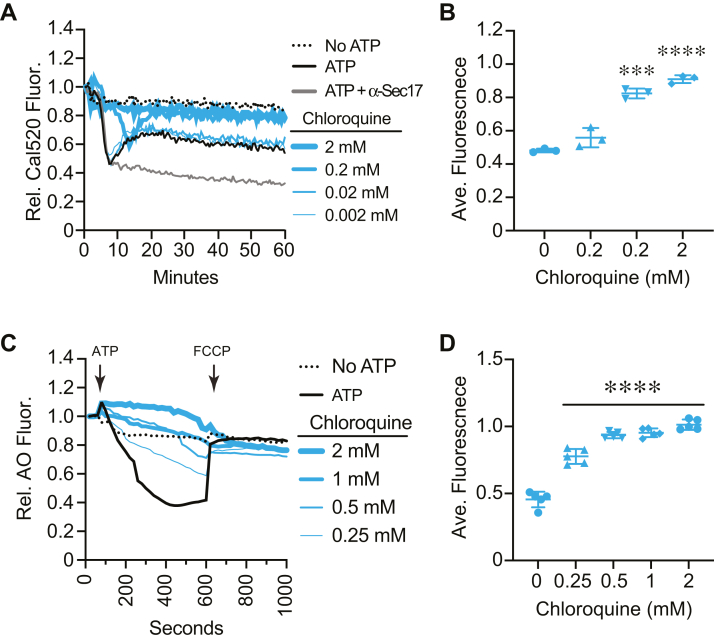
**Vacuole acidification affects Ca**^**2+**^**transport.***A*, Ca^2+^ transport was measured using Cal-520 fluorescence assays using WT vacuoles in the presence or absence of ATP or ATP with a concentration curve of chloroquine. *B*, quantitation of multiple experiments in panel (*A*) showed a significant effect of treating reactions with chloroquine [F(8,18) = 18.61; ∗∗∗∗*p* < 0.0001] (One way ANOVA for multiple comparisons with no treatment as a control). Error bars are mean ± SD. Dunnett multiple comparison test was used for individual *p* values (n = 3). ∗∗∗*p* < 0.001, ∗∗∗∗*p* < 0.0001. *C*, AO fluorescence using WT vacuoles in the presence of a concentration curve of chloroquine. FCCP was added after 600 s to collapse the H^+^ gradient. *D*, quantitation of multiple experiments in panel (*C*) showed a significant effect of treating reactions with chloroquine-treated reactions [F(4,20) = 131.8; ∗∗∗∗*p* < 0.0001] (One way ANOVA for multiple comparisons with no treatment as a control). Error bars are mean ± SD. Dunnett multiple comparison test was used for individual *p* values (n = 5). ∗∗∗∗*p* < 0.001. AO, acridine orange; FCCP, carbonyl cyanide-4-(trifluoromethoxy) phenylhydrazone.

To start, we used WT vacuoles in the Cal-520 fluorescence assay to track the effects of vacuole acidification on Ca^2+^ transport. To do this, we treated vacuoles with chloroquine (CQ), which raises the pH of vacuoles (66, 67). Untreated control vacuoles took up Ca^2+^ as seen by the loss of Cal-520 fluorescence (Fig. 8, *A* and *B*). This was followed by a rise in fluorescence near the 15 min mark as a reporter for SNARE-dependent efflux. The anti-Sec17 IgG-treated vacuoles took up Ca^2+^ but did not release it later due to the lack of SNARE pairing. We found that Ca^2+^ transport was delayed with 250 μM CQ and completely blocked with higher concentrations. This indicated that acidified vacuoles were needed for optimal Ca^2+^ uptake. To confirm that the levels of CQ used sufficiently blocked vacuole acidification, we used it in the AO acidification assay. As expected, we observed that CQ inhibited vacuole acidification in a dose-dependent manner (Fig. 8, *C* and *D*).

**Figure 9:**
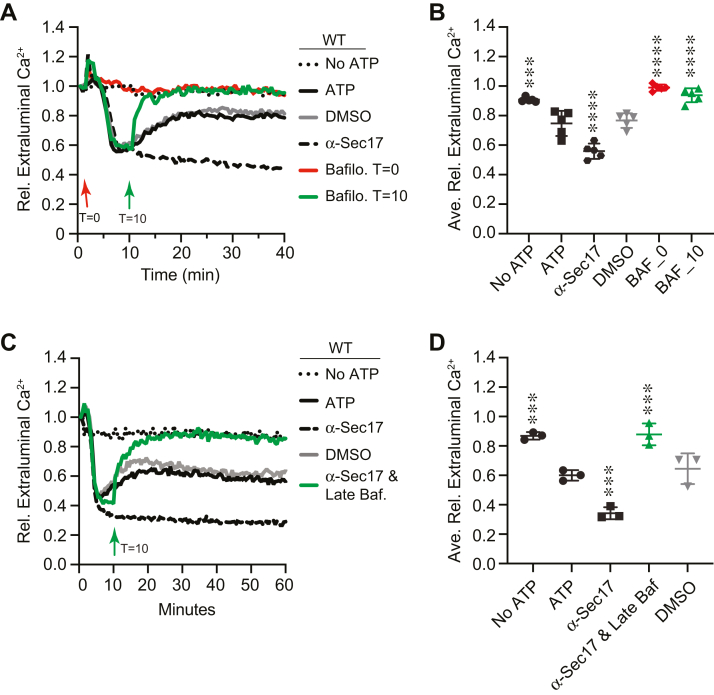
**V-ATPase inhibition affects Ca**^**2+**^**transport.***A*, Cal-520 fluorescence was measured using WT vacuoles in the presence or absence of 100 nM bafilomycin A1, 140 μg/ml anti-Sec17, and DMSO. Bafilomycin was added at the beginning (T = 0) or after 10 min of incubation (T = 10). *B*, quantitation of fluorescence at 20 min of multiple experiments in panel (*A*) showed a significant effect of treating reactions with anti-Sec17, DMSO, and bafilomycin added at T = 0 min and T = 10 min [F(5,24) = 49.44; ∗∗∗∗*p* < 0.0001] (One way ANOVA for multiple comparisons). Error bars are mean ± SD. Tukey’s multiple comparison test was used for individual *p* values (n = 5). ∗∗*p* < 0.01, ∗∗∗*p* < 0.001, ∗∗∗∗*p* < 0.0001. *C*, Cal-520 fluorescence was measured with WT vacuoles. Here, bafilomycin was added to reactions pretreated with anti-Sec17 to block SNARE activity. *D*, quantitation of fluorescence at 20 min of multiple experiments in panel (*C*) showed a significant effect of treating reactions with anti-Sec17, DMSO, and bafilomycin at T = 10 min to anti-Sec17 treated reactions [F(6,14) = 36.55; ∗∗∗∗*p* < 0.0001] (One way ANOVA for multiple comparisons). Error bars are mean ± SD. Tukey’s multiple comparison test was used for individual *p* values (n = 3). ∗∗*p* < 0.01, ∗∗∗*p* < 0.001, ∗∗∗∗*p* < 0.0001. DMSO, dimethyl sulfoxide.

While CQ served as a tool for raising the overall pH of the vacuole lumen, it is not a specific inhibitor of V-ATPase activity. In fact, CQ has been used to lower the pH of hyperacidified vacuoles lacking the Na^+^K^+^/H^+^ antiporter Nhx1 (66, 68). To test the effects of directly inhibiting V-ATPase activity, we used bafilomycin A1 in the Ca^2+^ flux assay. Adding bafilomycin at the beginning of the experiment completely blocked Ca^2+^ uptake by vacuoles, while the DMSO solvent had no effect, indicating that the effect was due to inhibiting V-ATPase activity (Fig. 9, *A* and *B*). Similarly, adding the V-ATPase inhibitor concanamycin A to reactions at the beginning completely blocked Ca^2+^ uptake (not shown). To see how quickly stopping V-ATPase function affected Ca^2+^ retention, we added bafilomycin after 10 min of incubation. This caused a rapid release of Ca^2+^ that reached the starting levels as measured by Cal-520 fluorescence. The Ca^2+^ release was faster and more pronounced than the natural SNARE-dependent release. Again, adding DMSO at the 10 min mark had no effect on Ca^2+^ flux, demonstrating that the release in Ca^2+^ was due to bafilomycin.

Because the release of Ca^2+^ after the initial uptake has been linked to *trans*-SNARE pairing, we wanted to see if the effects of bafilomycin were also SNARE dependent. To this end, we added bafilomycin to reactions that were pretreated with anti-Sec17 IgG. We found that it led to the release of Ca^2+^ with the same kinetics and magnitude as adding bafilomycin alone, indicating that the release was independent of SNARE complex formation (Fig. 9, *C* and *D*). We should note that bafilomycin inhibits SNARE pairing on its own when added early in the pathway; however, the effects of adding it during docking/tether on already formed SNARE complexes is unknown (49). It is worth remembering that blocking SNARE function does not affect the acidification of vacuoles as measured by AO fluorescence (41).

**Figure 10:**
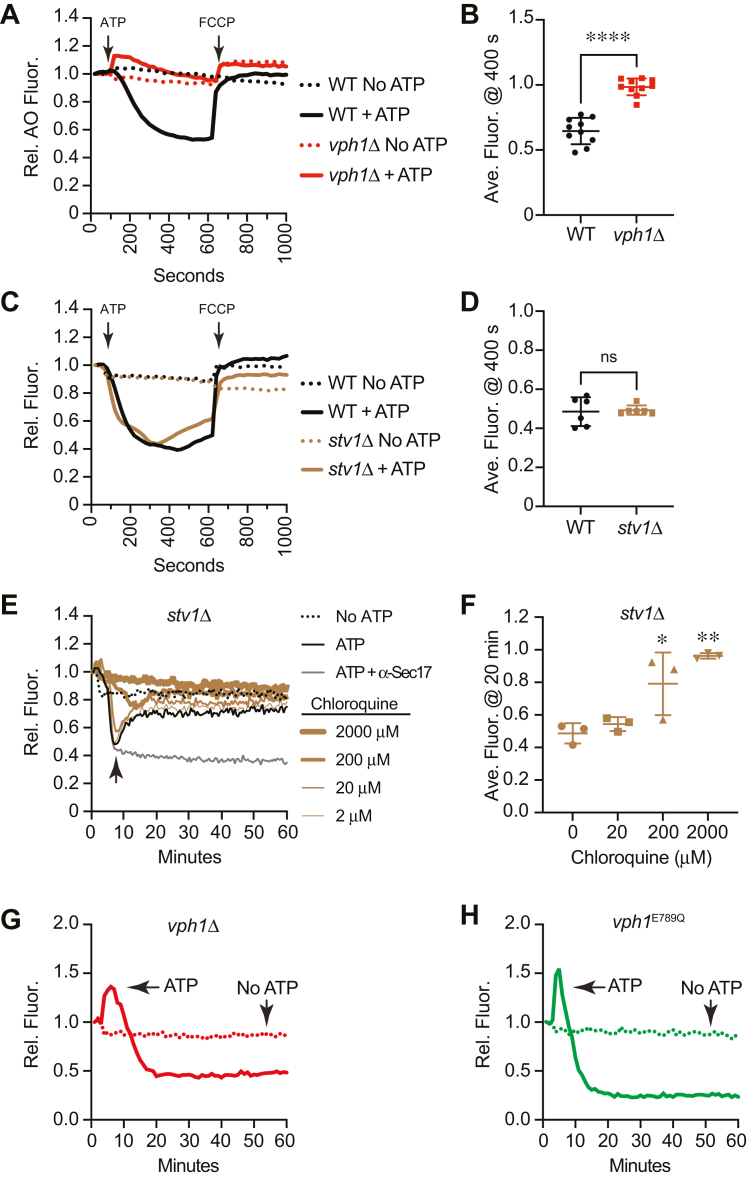
**V-ATPase function affects Ca**^**2+**^**transport.***A*, AO fluorescence using WT and *vph1*Δ vacuoles in the presence or absence of ATP. FCCP was added after 600 s to collapse the H^+^ gradient. *B*, average fluorescence at 400 s was compared between strains. Error bars are mean ± SD. (n = 9). Data were analyzed using Student’s unpaired two-tailed *t* test. ∗∗∗∗*p* < 0.0001 *C*, AO fluorescence using WT and *stv1*Δ vacuoles in the presence or absence of ATP. FCCP was added after 600 s to collapse the H^+^ gradient. *D*, average fluorescence at 400 s was compared between strains. Error bars are mean ± SD. (n = 6). *E*, Ca^2+^ transport assay using WT and *stv1*Δ vacuoles in the presence or absence of ATP or with both ATP and anti-Sec17 IgG to block SNARE-mediated Ca^2+^ efflux. *F*, quantitation of multiple experiments in panel (*E*) showed a significant effect of treating reactions chloroquine [F(3, 8) = 13.67; ∗∗*p* = 0.0016] (One way ANOVA for multiple comparisons with no treatment as a control). Error bars are mean ± SD. Dunnett multiple comparison test was used for individual *p* values (n = 3). ∗*p* < 0.05, ∗∗*p* < 0.01. *G*, Ca^2+^ transport assay using *vph1*Δ vacuoles in the presence or absence of ATP. *H*, Ca^2+^ transport assay using *vph1*^E789Q^ vacuoles in the presence or absence of ATP. AO, acridine orange; FCCP, carbonyl cyanide-4-(trifluoromethoxy) phenylhydrazone.

Finally, we asked how mutations of the V-ATPase complex would affect Ca^2+^ transport. To do this, we tested the effect of deleting *VPH1*. First, we used *vph1*Δ vacuoles in the AO fluorescence assay to verify that the deletion indeed inhibited vacuole acidification. We observed that AO fluorescence at 520 nm was not affected when using *vph1*Δ vacuoles, indicating that V-ATPase was blocked as shown previously (41) (Fig. 10, *A* and *B*). This also indicated that the Stv1 isoform of Vph1 does not replace Vph1 to form a functional V-ATPase. This is important to keep in mind because only the overexpression of Stv1 can partially restore vacuole quinacrine staining in cells lacking Vph1 (69). To verify that Stv1 does not affect vacuole acidification, we used *stv1*Δ vacuoles in the AO fluorescence assay. As shown in Figure 10, *C* and *D*, *stv1*Δ vacuoles were able to become acidified, as well as the WT. The *stv1*Δ vacuoles were as sensitive to CQ as compared to WT (not shown). This further acknowledges the requirement for Vph1 in vacuole acidification.

Next, we tested the effects of deleting *VPH1* and *STV1* on Ca^2+^ transport. We first tested *stv1*Δ vacuoles and found that they transported Ca^2+^ in a manner indistinguishable from WT vacuoles (Fig. 10, *E* and *F*). Moreover, they were equally sensitive to CQ treatment. When we tested *vph1*Δ vacuoles in Ca^2+^ transport assays, we found that they were able to take up Ca^2+^ but were inhibited in Ca^2+^ efflux (Fig. 10*G*). This showed that a functional V-ATPase was not needed for Pmc1 and Vcx1 activity. That said, the uptake was significantly slower *versus* the WT. Vacuoles lacking Vph1 did not plateau in Ca^2+^ uptake until ∼20 min of incubation compared with the 10 min uptake seen with WT. The uptake of Ca^2+^ by *vph1*Δ vacuoles appears at first to contradict the effects of bafilomycin; however, we must remain cognizant that physical interactions occur between Pmc1 and Vph1. We previously found that Pmc1 and Vph1 exist in a complex with the R-SNARE Nyv1 (16). Because Pmc1 activity is inhibited by its interaction with Nyv1 (70) it is possible that Vph1 also inhibits Pmc1 through being part of the complex. This interaction would not be disrupted by bafilomycin as it binds the c-ring. Therefore, we propose that acidification is not required for Ca^2+^ uptake.

The lack of an overall effect on Ca^2+^ uptake is also in disagreement with another study showing that the V_1_ subunit Vma2 was required for Ca^2+^ uptake *in vivo* (20). We hypothesized that this was due to the loss of Vma2 ATPase activity and overall V-ATPase function, while Vph1 remained present to interact with Pmc1. It is also possible that the effects of deleting *VMA2* eliminated interactions between V-ATPases and other proteins that could have direct or indirect effects on Ca^2+^ uptake. For instance, Vma2 interacts with actin, the formin Bni1, and the WASP homolog Las17 (71, 72, 73) and can stabilize filamentous actin in Arabidopsis (74). The state of actin polymerization itself has been shown to affect plasma membrane localized Ca-ATPases (75). While the links between Vma2, actin, and the vacuolar Ca-ATPase Pmc1 have not been well established, it is not unreasonable to think that different mutations in the V-ATPase could have distinct effects on Ca^2+^ uptake.

Unlike Ca^2+^ uptake, Ca^2+^ efflux was completely abolished in *vph1*Δ vacuoles, showing that a functional V-ATPase was needed for Ca^2+^ efflux. This is consistent with another study showing that anti-Vph1 IgG both delayed Ca^2+^ uptake and blocked downstream efflux (76). We must also note that *vph1*Δ vacuoles consistently show a release of Ca^2+^ upon addition of ATP. The release lasts nearly 10 min before the vacuoles take up Ca^2+^. This accounts for the overall delay in Ca^2+^ uptake. The reason for this is unknown. We do know that Ca^2+^ is instantly released upon ATP addition when both Pmc1 and Vcx1 are deleted (16). This is attributed to a constant ATP-dependent release of Ca^2+^ that is masked or inhibited when both uptake mechanisms are in place; however, the source of the Ca^2+^ release remains unknown. We conclude that the V-ATPase has a dual function maintaining Ca^2+^ transport homeostasis. One function depends on the inhibition of Pmc1 by Vph1 and the second is the need for an H^+^ gradient for Ca^2+^ efflux.

The lack of Vph1 is known to destabilize the V_1_–V_O_ holocomplex, which raises the question of whether Ca^2+^ transport would be affected by a complete but attenuated V-ATPase. To test this, we used the *vph1*^E789Q^ point mutant that allows for the assembly of the V-ATPase but inhibits its function in a *vph1*Δ *stv1*Δ double deletion (77). We used *vph1*^E789Q^ in the single *vph1*Δ deletion background since we already found that deleting *STV1* had no effect on Ca^2+^ transport. We found that *vph1*^E789Q^ vacuoles released Ca^2+^ at the start of the assay, as did the *vph1*Δ vacuoles (Fig. 10*H*). However, the duration of the release was short in comparison and total Ca^2+^ uptake was completed by the 10 min mark as seen with WT vacuoles. Also, similar to the Vph1 deletion, *vph1*^E789Q^ vacuoles did not release Ca^2+^. These data suggest that the assembly of the V_1_–V_O_ blocks the inhibitory effects of Vph1 on Pmc1, perhaps through inducing conformational changes.

## Discussion

In this study, we present data showing that Ca^2+^ transport and vacuole acidification are interdependent and that they can be affected by PI(3,5)P_2_ under specific conditions. Elevating PI(3,5)P_2_ levels through the hyperactive *fab1*^*T2250A*^ mutation increases vacuole acidification, whereas the kinase-dead *fab1*^*EEE*^ mutant reduces acidification. Similarly, the presence or absence of PI(3,5)P_2_ has opposing effects on Ca^2+^ transport. *In vitro* Ca^2+^ transport assays show that Ca^2+^ is taken into the vacuole lumen upon the addition of ATP, after which a wave of Ca^2+^ is released from the organelle. While the uptake is independent of SNAREs, the release requires the formation of *trans*-SNARE complexes between paired vacuoles (14). In a previous study, we found that either adding exogenous C8-PI(3,5)P_2_ or expressing *fab1*^*T2250A*^ blocked the net Ca^2+^ released upon *trans*-SNARE complex formation between vesicles (16). On the other hand, blocking Fab1 activity with apilimod arrested Ca^2+^ uptake and elicits the accelerated release of Ca^2+^. These findings led to the hypothesis that V-ATPase activity and Ca^2+^ transport are linked in part through the production of PI(3,5)P_2_.

We further demonstrated the connection between H^+^ and Ca^2+^ transport by testing the effect of changing the concentration of free Ca^2+^ on the formation of an H^+^ gradient. First, we showed that increasing extraluminal Ca^2+^ blocked the shift in AO fluorescence, indicating that the H^+^ gradient had been broken. This was linked to the role of the Ca^2+^/H^+^ exchanger Vcx1. The converse was seen when extraluminal Ca^2+^ was chelated with EGTA. The lack of free Ca^2+^ augmented the rate of H^+^ uptake and formation of a proton gradient. Interestingly, the deletion of *VCX1* does not replicate the accelerated H^+^ uptake seen with EGTA, suggesting that the difference was not due to the lack of H^+^ export by Vcx1. Thus, we can postulate that a separate Ca^2+^-dependent mechanism could modulate vacuole acidification.

### Ca^2+^ transporters interact with the V-ATPase

Based on several reports looking at mammalian counterparts, physical interactions between V-ATPase subunits and various Ca^2+^ transporters are not rare. For instance, L-type Ca^2+^ channels in murine cells physically interact with the G2 subunit of the V-ATPase, and inhibition of V-ATPase function leads to the mislocalization of the Ca^2+^ channel (78). Others have reported that the R-type Cav2.3 Ca^2+^ channel interacts with the G1-subunit of the V-ATPase and showed that bafilomycin A1 reduces Ca^2+^ transport (79). In yeast, the addition of an antibody against Vph1 blocks Ca^2+^ efflux (76). While not shown directly, it is possible that the antibody against Vph1 physically interfered with the interaction between the V-ATPase and the Ca-ATPase Pmc1. Previously, we found that Pmc1 physically interacts with a protein complex that includes Vph1 and the SNARE Nyv1 (16, 70). In this study, we found that deleting *VPH1* does not inhibit Ca^2+^ uptake, while efflux was abolished. On the other hand, when V-ATPase activity was inhibited with bafilomycin A1, both uptake and efflux were inhibited. So, what is the difference? The difference is the presence or absence of Vph1. We know that Vph1 binds to Pmc1 and Nyv1, and we know that Nyv1 inhibits Pmc1 activity. Therefore, it stands to reason that Vph1 has an inhibitory effect on Pmc1. This is likely why we see Ca^2+^ uptake in *vph1*Δ vacuoles that are otherwise unacidified. Taken together, we think that acidification and physical inhibition by Vph1 can be separated as modes of regulating Ca^2+^ flux. It is also apparent that the physical interaction of transporters is likely a major component of their coregulation.

The fact that the V-ATPase performs functions that are in addition to H^+^ translocation has been shown by many studies. For instance, in *Drosophila* neurons, the a1 subunit V100 (yeast Vph1) of the V_O_ complex interacts with Ca^2+^-loaded CaM to promote normal eye development (80). In mammalian cells, the V-ATPase recruits the small GTPase Arf6 and its nucleotide exchange factor ARNO from the cytosol to endosomal membranes through interaction with the c-ring and a-2 subunits, respectively (81). These interactions play a role in the endolysosomal degradative pathway. In neurons, the c-ring binds to the SNARE Synaptobrevin to reduce neurotransmitter release through SNARE-dependent fusion of synaptic vesicles and the plasma membrane (82). Finally, in osteoclasts, the d2 subunit promotes osteoclast fusion independent of pH changes caused by V-ATPase (83). Many more examples like these exist to illustrate that the V-ATPase can physically interact with other proteins to affect a variety of pathways.

### How does Ca^2+^-dependent signaling play a role in linking Ca^2+^ transport with vacuole acidification?

Based on the literature, it is almost certain that Ca^2+^ is activating CaM signaling as part of vacuole acidification. First, CaM itself was shown to play a role in vacuole fusion (84). In that study, CaM was inhibited with antibodies or with specific inhibitors and found that the activity was mostly after the docking stage, which is ∼20 min into the pathway. On the other hand, vacuole acidification is complete after ∼6 min, suggesting that any effect that CaM has on acidification would likely be independent from what affects fusion. Our preliminary studies show that the CaM inhibitor W7 affects vacuole acidification by an unknown mechanism (our unpublished results). Second, the CaM-dependent protein phosphatase Calcineurin is known to inhibit Vcx1 even though its direct dephosphorylation has not been shown (7). Taken together, it is likely that CaM-dependent signaling is important in generating the vacuolar H^+^-gradient. Future studies will be needed to address this link.

In conclusion, this study shows that vacuole acidification by the V-ATPase is regulated by Ca^2+^ homeostasis, which itself is affected by the V-ATPase. While the mechanism(s) for this relationship remains to be elucidated, we can add that their interdependence on the vacuole could be associated with the production of PI(3,5)P_2_. These connections begin to unveil a more complicated network of interactions that integrates the composition of the membrane with ion homeostasis.

## Experimental procedures

### Reagents

Soluble reagents were dissolved in Pipes-Sorbitol buffer (20 mM Pipes–KOH, pH 6.8, 200 mM sorbitol) with 125 mM KCl, unless indicated otherwise. C8-PI(3,5)P_2_ (1,2-dioctanoyl-phosphatidylinositol 3,5-bisphosphate) was purchased from Echelon Inc. ATP was purchased from RPI. Apilimod, bafilomycin A1, concanamycin A, and verapamil were from Cayman Chemical and dissolved in DMSO. AO, Calcofluor White solution, chloroquine, CoA, creatine kinase, EGTA, FCCP, and quinacrine were purchased from Sigma. Creatine phosphate was from Abcam. Cal-520 dextran conjugate molecular weight 10,000 was from AAT Bioquest. FM4-64 was purchased from ThermoFisher. Anti-Sec17 IgG (64), Pbi2 (Proteinase B inhibitor 2) (85), GST-ML1N (11), and GST-FYVE (38) were prepared as described and dialyzed against Pipes-Sorbitol buffer with 125 mM KCl.

### Strains and proton transport assay

Vacuoles were isolated as described from BJ3505 genetic backgrounds and used for vacuole acidification and Ca^2+^ transport assays (Table 1) (15, 16, 41, 86, 87). *STV1* was deleted by homologous recombination using PCR products amplified from pAG32 plasmid with primers 5′-STV1-KO (5’ – AGGCCCACGAAGGTGATTGGAAGTTCAGTGTTGAATCT GTTTAGCTTGCCTCGTCC – 3′) and 3′-STV1-KO (5′- GCAAACGTAGCGCATGCAACATTGCGTGGATGGCGGCGTTAGTATCGA – 3′), with homology flanking the *STV1* coding sequence. The PCR product was transformed into chemically competent yeast by standard lithium acetate methods and plated on yeast extract–peptone–dextrose (YPD) containing hygromycin (200 μg/ml) to generate BJ3505 *stv1::hyhMX4* (RFY108). RFY107 (*vph1*Δ) was transformed with pRS316-VPH1^E789Q^ (a gift from Dr P. Kane, Upstate Medical University, Syracuse, NY) to make RFY109. The proton pumping activity of isolated vacuoles was performed as described by others with some modifications (30, 51). *In vitro* acidification reactions (60 μl) contained 20 μg vacuoles, reaction buffer (20 mM Pipes-KOH pH 6.8, 200 mM sorbitol, 125 mM KCl, 5 mM MgCl_2_), ATP-regenerating system (1 mM ATP, 0.1 mg/ml creatine kinase, 29 mM creatine phosphate), 10 μM CoA, 283 nM Pbi2 (inhibitor of protease 2), and 15 μM of AO. Reaction mixtures were loaded into a black, half-volume 96-well flat-bottom plate with nonbinding surface. ATP-regenerating system or buffer was added, and reactions were incubated at 27 °C while AO fluorescence was monitored. Samples were analyzed in a fluorescence plate reader (POLARstar Omega, BMG Labtech) with the excitation filter at 485 nm and emission filter at 520 nm. Reactions were initiated with the addition of ATP-regenerating system following the initial measurement. After fluorescence quenching plateaus were reached, we added 30 μM FCCP to collapse the proton gradient and restore AO fluorescence.

**Table 1:** Yeast strains used in this study

Strain	Genotype	Source
BJ3505	*MAT*a *ura3–52 trp1-*D*101 his3-200 lys2–801 gal2 (gal3) can1 prb1-*D*1.6R pep4::HIS3*	(87)
RFY74	BJ3505, *yvc1::kanMX6*	(15)
RFY76	BJ3505, *fab1::kanMX6*	(15)
RFY78	BJ3505, *fab1::kanMX6*, *pRS416-FAB1*^*T2250A*^	(15)
RFY80	BJ3505, *fab1::kanMX6*, *pRS416-FAB1*^*EEE*^	(15)
RFY84	BJ3505, *pmc1::kanMX6*	(16)
RFY86	BJ3505, *vcx1::kanMX6*	(16)
RFY107	BJ3505, *vph1::hphMX4*	(41)
RFY108	BJ3505, *stv1::kanMX6*	This study
RFY109	BJ3505, *vph1::hphMX4*, *pRS316-VPH1*^*E789Q*^	This study

### Calcium transport

Vacuolar Ca^2+^ transport was measured as described (53, 88, 89). *In vitro* Ca^2+^ transport reactions (60 μl) contained 20 μg vacuoles from BJ3505 backgrounds, reaction buffer, 10 μM CoA, 283 nM Pbi2, and 150 nM of the Ca^2+^ probe Cal-520 dextran conjugate molecular weight 10,000. Reaction mixtures were loaded into a black, half-volume 96-well flat-bottom plate with nonbinding surface. ATP-regenerating system was added, and reactions were incubated at 27 °C while Cal-520 fluorescence was monitored. Samples lacking ATP were used a negative control for Ca^2+^ uptake. Antibody against the SNARE cochaperone Sec17 was added as a negative control for SNARE-dependent Ca^2+^ efflux (14). Samples were analyzed using a fluorescence plate reader with the excitation filter at 485 nm and emission filter at 520 nm. Reactions were initiated with the addition of ATP-regenerating system following the initial measurement. The effects of inhibitors on efflux were determined by the addition of buffer or inhibitors immediately following Ca^2+^ influx. Calibration was done using buffered Ca^2+^ standards (Invitrogen).

### Fluorescence microscopy

*In vivo* vacuole staining with quinacrine and FM4-64 (5 μM) was carried out as described (88, 90, 91). WT BJ3505 cells were grown overnight in YPD broth and diluted with fresh YPD to an *A*_600_ of ∼0.6 to 0.8. The new YPD was buffered to pH 7 with 50 mM Tris–HCl, pH 7.5. First, cells were treated with either buffer or CaCl_2_ and incubated for 15 min at 30 °C, after which cells were stained with 200 μM quinacrine and incubated for an additional 20 min at 30 °C. Cells were harvested by centrifugation, washed once with PBS, pH 7.2, and resuspended in 20 μl PBS. Cell walls were stained by adding 10 μl of Calcofluor White solution and incubating for 2 min. Cell samples were mixed with low-melting agarose and mounted onto glass slides and examined by fluorescence microscopy. Images were acquired using a Zeiss Axio Observer Z1 inverted microscope equipped with an X-Cite 120XL light source, Plan Apochromat 63X oil objective (NA 1.4), and an AxioCam CCD camera. Quinacrine was visualized using a 38 HE EGFP shift-free filter set, FM4-64 was visualized with a 42 HE CY 3 shift-free filter set, and Calcofluor White was visualized with a 49 4′,6-diamidino-2-phenylindole shift-free filter set.

## Data analysis and statistics

Results are expressed as the mean ± SD. Experimental replicates (n) are defined as the number of separate experiments. Statistical analysis was performed by Student’s unpaired two-tailed *t* test or one-way ANOVA for multiple comparisons using Prism 9 (GraphPad). Statistical significance is represented as follows: ∗*p* < 0.05, ∗∗ *p* < 0.01, ∗∗∗ *p* < 0.001, ∗∗∗∗ *p* < 0.0001.

## Data availability

All primary data are available upon request. Additional data sharing information is not applicable to this study.

## Conflict of interest

The authors declare that they have no conflicts of interest with the contents of this article.
